# MAPK phosphatase 5 inhibits IRF3

**DOI:** 10.18632/oncotarget.5096

**Published:** 2015-08-03

**Authors:** Chin Wen Png, Yongliang Zhang

**Affiliations:** Department of Microbiology, Yong Loo Lin School of Medicine, and Immunology Programme, Life Science Institute, National University of Singapore, Singapore

The interferon regulatory factor 3 (IRF3) is a transcriptional factor known to be essential for the expression of type I interferons (IFNs) including IFNα and IFNβ. Type I IFNs bind to the ubiquitously expressed IFNα receptors (IFNAR), leading to the induction of more than 300 IFN-stimulated genes (ISGs) to establish the antiviral status of the cells [1]. Although IRF3-type I IFN response is critical for immune defences against viral infection, excessive type I IFNs is deleterious to the host. Dysregulated type I IFN responses are associated with inflammatory and autoimmune diseases such as systemic lupus erythematosous and Aicardi-Goutieres syndrome [2]. Therefore, tightly regulated IRF3-type I interferon activation is important for effective defences against viral pathogens and prevention of type I interferonopathies.

IRF3 is constitutively expressed in various cell types and is localized in the cytoplasm in an inactive state without infection. In response to virus infection, specific serine residues in the C-terminal regions of IRF3 including Ser396, Ser405, and Ser386 are phosphorylated by its upstream activators such as TBK1, which leads to IRF3 dimerization and nuclear translocation for gene induction [3]. However, how IRF3 activation is negatively regulated is not well understood.

In a recent study published in *Cell Reports*, we identified MAPK phosphatase 5 (MKP5), a member of the dual-specificity phosphatase family, as a negative regulator of IRF3-type I interferon axis [4]. We showed that MKP5 protein binds to a C-terminal region of IRF3 and inactivate IRF3 by dephosphorylating Ser396 and Ser386 residues. The inactivation of IRF3 by MKP5 is dependent on MKP5 phosphatase activity or its binding to IRF3. This is confirmed since MKP5 phosphatasedeficient mutant is unable to dephosphorylate IRF3 and MKP5 mutant lacking IRF3-binding motifs fails to suppress IRF3 nuclear translocation upon virus infection. In addition, the negative regulation of IRF3 activation by MKP5 is independent of its interaction with mitogenactivated kinases (MAPKs), as MKP5 mutant deficient in MAPK-binding is still able to suppress IRF3 nuclear translocation.

The *in vitro* biochemical observations on the role of MKP5 as a negative regulator of IRF3 were substantiated by *ex vivo* and *in vivo* evidences. In macrophages and dendritic cells, the expression of MKP5 at both mRNA and protein levels was induced upon infection by viruses including influenza virus, vesicular stomatitis virus (VSV) and sendai virus (SeV). Compared to wildtype (WT) cells, MKP5 knockout (KO) cells have increased IRF3 phosphorylation, nuclear translocation and accumulation followed infection by these RNA viruses. Consequently, the expression of type I IFNs, including IFNα and IFNα, as well as various ISGs such as ISG15, OAS, RANTES and Viperin is increased in MKP5 KO cells. Such enhanced type I IFN response in macrophages is associated with reduced influenza viral protein expression and virus replication upon H1N1 influenza infection. Increased IRF3-type I IFN response and reduced virus replication were also observed in the lungs from MKP5 KO mice compared with WT mice in response to H1N1 influenza virus infection. MKP5 KO mice also developed less severe disease upon infection with influenza and VSV. Furthermore, MKP5 deficient mice have better survival upon lethal dosage of H1N1 influenza infection compared to WT mice. Collectively, these data shows that MKP5 is a phosphatase that directly dephosphorylates IRF3 for negative regulation of the IRF3-type I IFN response.

Interestingly, we found that the non-structural protein 1 (NS1) of influenza virus is important for the induction of MKP5 expression in macrophages. NS1 protein of influenza virus plays a central role in virus replication and blockade of host innate immunity [5]. Therefore, induction of MKP5 expression could be a prosurvival strategy employed by the viruses during infection. At the same time, MKP5 could be a strategy of the host to regulate the intensity of antiviral immune response to avoid uncontrolled immunopathology. In all, our study have clearly demonstrated a novel regulatory role of MKP5 in IRF3-type I response. Further investigation is ongoing to examine the beneficial effect of MKP5 to the host in other immune-mediated pathology.
